# On the Use of Kermes Mineral in Diseases of the Respiratory Organs

**Published:** 1846-08

**Authors:** 


### *On the use of Kermes Mineral in Diseases of the Respiratory Organs. By* Dr. Herpin, *of Geneva.*

Dr. Herpin has diligently observed the effects of this medicine during eight years, and has arrived at some interesting conclusions respecting it. If we consult dictionaries, dispensatories, &c., we find this substance stated as being chiefly indicated in chronic and suffocative catarrh, humid asthma, and at the termination of pertussis and pneumonia, especially in aged persons. Dr. Herpin reports differently. He says that he has never seen its use followed by even temporary benefit in the latter stages of *pneumonia* in the aged, when the mucous *rales* are abundant and asphyxia imminent. Given, too, at the commencement of pneumonia following capillary bronchitis, it is far inferior to tartar-emetic: and alterative doses of this same remedy are also far superior to it in the capillary *bronchitis* of old persons and children. The same want of success attended its trials in asthma and pertussis.

But when the disease, instead of being situated in the parenchyma and smaller bronchi, occupies the *larger passages* the result is very different. Laennec and all those who have come after him have repeated the erroneous statement, that *bronchitis*, from its very commencement, when a mere coryza, imparts to the ear a loud *rale*. Dr. Herpin some time since showed that bronchitis at the upper part of the tube, accompanied by considerable secretion, gave no ascultatory sign whatever. It is in these cases, where no abnormal sound exists, that *Kermes* is so useful, and is most so in the acute stage. He does not mean to say that it will arrest the progress of every pulmonary disease beginning without auscultatory signs, for hooping-cough, pneumonia and phthisis are among such. The catarrhs commencing at the upper part of the tube are soon arrested; but if *rales* announce the supervention of deep-seated bronchitis, other medicines must be resorted to. In *tracheitis*, (denoted by pain opposite the top of the sternum, sometimes difficult deglutition, a hoarse, tearing paroxysmal cough and hoarseness, ) the Kermes is still more indicated. No other medicine produces so rapid an effect in *laryngitis*, a few ¼ grain doses often removing the hoarseness in a few hours, when the disease is recent. In this way it is of great service to singers. *False-croup* is very advantageously treated by it, and if seen early enough, it may of good service in the true disease. *Chronic laryngitis*, when not dependent upon phthisis, may be benefitted by this medicine, but in an inverse proportion to its duration. Even when it does not cure it (for relapse is very frequent) it give at least great relief. In the only case of *thymous asthma*, Dr. H. has had an opportunity of trying it, and which was a very bad one, it succeeded completely. In affections of the *pharynx* no success followed the use of the medicine, unless indeed these were connected with disease of the larynx. A frequent cause of *deafness* is a catarrhal condition of the extremity of the Eustachian tube, and in this case the Kermes effects a cure if the deafness has not existed beyond some weeks, and even alleviates it frequently when of very old standing. From his observations, Dr. H. believes he may deduce the conclusion, that *Kermes is to some extent a specific for the affections of the upper portions of the air-passages.*

The *dose* has varied from 1 to 12 grs. in the 24 hours. M. H. has never exceeded the latter quantity, and, as a general rule, from 3 ot 6 grains suffice. If we except infants less than 1 or 2 years old, the dose need not be much varied on account of age, children tolerating the remedy almost as well as adults. It may be given in an emulsion, powder with sugar, lozenges or pills. At the commencement of the affection, or when the respiration is much oppressed, it is desirable to excite vomitiug. Three grains will certainly effect this, as will sometimes one or two in adults, and half a grain in children. To avoid purging or vomiting, we should give only very small doses, and after meals. When tolerance is once established, the Kermes does not irritate the stomach again. When first given it causes a sense of heat and dryness in the throat, which soon become relieved by an increased humidity and expectoration. Dr. Lombard, who has employed this medicine with excellent effects, has often observed rose-colored streaks in the expectoration; but these soon disappear.—*Medico-Chirurgical Review.*

